# Evaluation of Efficacy and Review of Side Effect Profile in Epilepsy Patients Treated with Extended-Release Levetiracetam

**DOI:** 10.12669/pjms.41.11.12553

**Published:** 2025-11

**Authors:** Zeynep Ozdemir, Fatma Aybuke Cokkacar, Fulya Eren, Gunay Gul

**Affiliations:** 1 Zeynep Ozdemir, University of Health Sciences Bakırköy Prof. Dr. Mazhar Osman Mental Health and Neurological Diseases Training and Research Hospital, Neurology Department İstanbul, Türkiye; 2 Fatma Aybuke Cokkacar, University of Health Sciences Taksim Training and Research Hospital, Neurology Department, İstanbul, Türkiye; 3 Fulya Eren, University of Health Sciences Taksim Training and Research Hospital, Neurology Department, İstanbul, Türkiye; 4 Gunay Gul, University of Health Sciences Bakırköy Prof. Dr. Mazhar Osman Mental Health and Neurological Diseases Training and Research Hospital, Neurology Department İstanbul, Türkiye

**Keywords:** Epilepsy, Levetiracetam Extended Release, Efficacy of LEV-ER, Side Effects of LEV-ER

## Abstract

**Background and Objective:**

Extended-release levetiracetam offers pharmacokinetic advantages over immediate-release formulations, including stable plasma concentrations and once-daily dosing, which may improve seizure control and reduce psychiatric side effects. This study aimed to evaluate the clinical efficacy, tolerability, and psychiatric side effect profile of LEV ER in epilepsy patients, either newly initiated or switched from LEV IR.

**Methodology:**

A retrospective multicenter cohort study was conducted at two tertiary neurology centers in Istanbul, Turkey. Medical records of 40 patients with epilepsy treated with LEV ER for 12–18 weeks were reviewed. Demographic data, seizure frequency, EEG/MRI findings, prior LEV use, treatment type, and psychiatric comorbidities were analyzed.

**Results:**

The cohort included 27 females and 13 males (mean age 37.6 years). Mean seizure frequency decreased from 1.66 to 0.6 per month after LEV ER initiation (p < 0.001). Of the 36 patients switched from LEV IR, 88.4% with psychiatric adverse effects showed clinical improvement. Among four patients who initiated LEV ER as first-line therapy due to a preference for once-daily dosing, two discontinued treatment because of adverse effects. Clinical improvement was observed in the complaints of 83.3% of patients who switched from LEV IR to ER form due to psychiatric medication use.

**Conclusions:**

LEV ER was associated with a significant reduction in seizure frequency and demonstrated a favorable tolerability profile, particularly with respect to psychiatric adverse effects. Transitioning from LEV IR to LEV ER may offer clinically meaningful advantages in selected patient populations. Nevertheless, further prospective studies with larger cohorts are warranted to confirm these findings.

## INTRODUCTION

Epilepsy is a chronic neurological disorder characterized by recurrent, unprovoked seizures that affect millions of people worldwide. Over the past three decades, there has been a notable increase in the number of licensed anti-seizure medications (ASMs). Currently, more than 30 ASMs are available for the treatment of epilepsy. In addition to these drugs, the development of extended-release (ER) delivery systems has significantly enhanced treatment options by increasing their efficacy, reducing side effects, and improving patient compliance.^1^ Levetiracetam is a particularly efficacious and well-tolerated treatment. Levetiracetam is classified as a second-generation ASMs and its mechanism of action involves binding to synaptic vesicle protein 2A.^2^ It is extensively distributed in the central nervous system (CNS).

The precise mechanism of action of levetiracetam remains unclear. However, current evidence indicates that levetiracetam may possess the ability to selectively prevent the spread of hyper synchronization of epileptic activity. This drug inhibits epileptiform activity without affecting normal neuronal excitability. The extended-release form of levetiracetam (LEV ER) offers the advantage of a single-day dosing, which provides constant plasma concentrations over the course of treatment.^1^ This represents a significant advancement in epilepsy treatment. The use of the immediate-release form of levetiracetam (LEV IR [immediate release]) rapidly achieves plasma concentrations, thereby providing a faster onset of action.

Nonetheless, an abrupt increase in plasma concentrations may precipitate adverse psychiatric effects in some patients, including agitation, irritability, depression, and anxiety.^3^ In clinical trials, the proportion of patients receiving LEV IR exhibited a higher incidence of psychiatric side effects during treatment. Levetiracetam ER provides slower and more constant absorption of the drug, resulting in less fluctuation in plasma concentrations. This feature may reduce the side effects of sudden plasma peaks. Some studies have reported a lower frequency and severity of psychiatric side effects (particularly agitation and irritability) in patients taking LEV ER.^4^ Considering the evidence presented above, it was postulated that LEV ER would prove to be an efficacious and well-tolerated therapeutic option for patients with epilepsy.^5^

The aim of this study was to investigate and compare the effects of clinical data from patients using LEV ER for the first time or switching from the LEV IR to the LEV ER formulation on seizure frequency, adverse effects, and treatment compliance.

## METHODOLOGY

### Data sources and search strategy:

The file data of patients who were followed up with a diagnosis of epilepsy for at least one year according to the revised diagnostic criteria of the International League Against Epilepsy (ILAE) 2025 revised diagnostic criteria by the neurology outpatient clinics of two different centers were retrospectively analyzed. This multicenter study was conducted with patients from the University of Health Sciences Bakırköy Prof. Dr. Mazhar Osman Mental Health and Neurological Diseases Training and Research Hospital and the University of Health Sciences Taksim Training and Research Hospital. Patients aged between 18 and 65 years who had been receiving extended-release levetiracetam treatment for a minimum duration of 12-18 weeks were included in the study cohort. Patients with incomplete data and those who had initiated psychiatric medication within the previous three months were excluded from the study. All participants were informed about the study procedures and provided written informed consent before inclusion. The demographic characteristics of the patients, including age, sex, age at disease onset, mean disease duration, seizure semiology, EEG and MRI findings, laboratory results (complete blood count and biochemistry tests), seizure frequency before and after LEV ER, and side effects reported by the patient were documented according to the file data. The frequency of seizures during patient follow-up was documented in the file in accordance with the data recorded in the seizure diaries maintained by the patients themselves or their caregivers.

### Ethical statement:

This study was conducted in accordance with the ethical principles of the Declaration of Helsinki. Ethical approval for the study was obtained from the Clinical Research Ethics Committee of Taksim Training and Research Hospital (Number: E-62190176-663.05-259626753, Date: November 15, 2024). This study was not supported by any funding source, and the authors declare that there is no conflict of interest.

### Data analysis:

Data pertaining to the patients were recorded using SPSS version 22, and normality distribution analysis was performed. Thereafter, purposeful comparisons were made using appropriate statistical methods in accordance with the results of the normal distribution.

## RESULTS

The study cohort comprised 27 female and 13 male patients with a mean age of 37.6 years (range, 19-65 years). The mean disease duration was 167.8 months (range, 12-627 months) and the mean seizure frequency prior to LEV ER administration was 1.66 per month. Following the administration of LEV ER, the frequency of seizures decreased significantly, from 1.66 per month before treatment to 0.6 per month post-treatment (p <0.001). Seizure type, EEG findings, MRI findings, prior administration of LEV IR, therapy type and the presence of psychiatric medication use in the study population are presented in Table-I.

**Table-I:** Clinical and treatment characteristics of study cohort.

Variable	Category	N (%)
Seizure type	Focal	10 (25.0)
	Generalized	30 (75.0)
EEG findings	Focal	11 (27.5)
	Generalized	11 (27.5)
	Normal	4 (10.0)
MRI findings	Pathologic	12 (30.0)
	Normal	28 (70.0)
Prior administration of LEV IR	Yes	36 (90.0)
	No	4 (10.0)
Therapy type	Monotheraphy	27 (67.5)
	Polytherapyh	13 (32.5)
Psychiatric medication	Yes	28 (70.0)
	No	12 (30.0)

Treatment was initiated directly with LEV ER in 4 patients due to preference for once-daily dosing. Among 36 patients switched from LEV IR to LEV ER, reasons included treatment compliance (n=8), sleep disturbances (n=2), and psychiatric adverse effects (n=26). Of the four patients who initiated LEV ER as first-line therapy due to a preference for once-daily dosing, two discontinued treatments because of adverse effects. Among those who were switched from LEV-IR to LEV-ER, clinical improvement was observed in 50% of patients with compliance-related issues, in all patients with sleep disturbances, and in 88.4% of those with psychiatric side effects.

**Fig.1:**
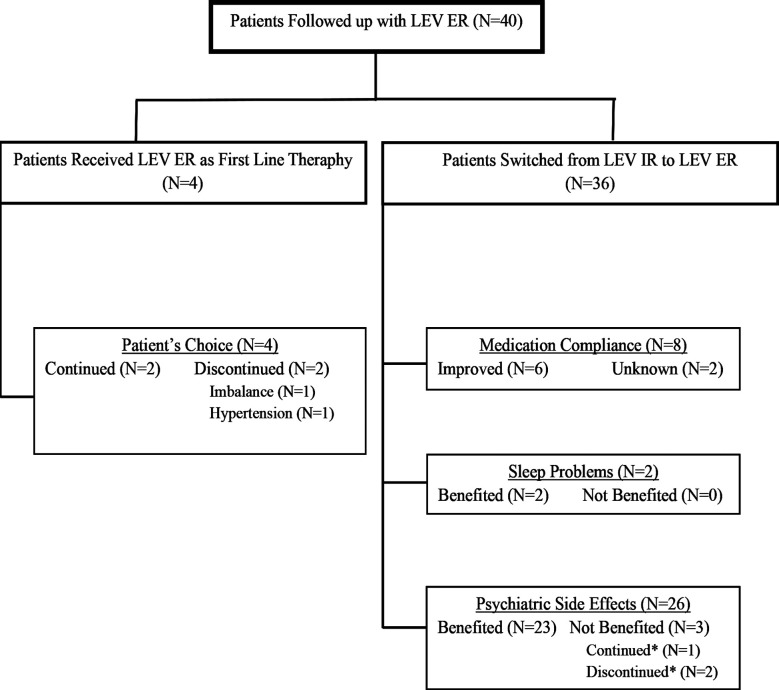
The treatment profiles of patients followed with LEV-ER and the clinical outcomes.

The treatment profiles of patients followed with LEV-ER and the clinical outcomes are presented in Fig.1.

## DISCUSSION

The extended-release formulation of levetiracetam was developed with the objective of providing patients with the convenience of once-daily dosing and potentially improving compliance and the efficacy-tolerability ratio. Studies conducted in both healthy volunteers and different ethnic groups have demonstrated that LEV ER tablets exhibit dose-proportional pharmacokinetic properties within the 500–3000 mg dose range.^6^,^7^ Comparative clinical studies evaluating IR and ER formulations of levetiracetam have generally reported similar outcomes with respect to seizure frequency. In the study by Joon Won Kang et al., which included 44 adolescent patients who were switched from LEV-IR to LEV-ER, no significant differences were observed in seizure frequency between the two formulations; however, treatment compliance was found to be better with the ER formulation.^8^ Likewise, a randomized, double-blind, multicenter trial comparing LEV-IR and LEV-ER in adults with partial epilepsy revealed that both formulations significantly reduced seizure frequency; notably, the LEV-ER group exhibited a higher rate of complete seizure freedom during the 12-week treatment period.^9^ Consistent with these findings, our study demonstrated a statistically significant reduction in self-reported seizure frequency following the initiation of LEV-ER.

A review on the clinical pharmacokinetics of levetiracetam highlighted that LEV-ER is bioequivalent to the IR formulation, enhances patient adherence through once-daily dosing convenience, is suitable for administration with food, and exhibits dose-proportional pharmacokinetic properties.^10^ In our cohort, four patients initiated treatment directly with the ER formulation based on a preference for once-daily dosing. Among these, two patients (50%) continued treatment successfully, while the other two discontinued due to adverse effects. Furthermore, of the eight patients who were switched from the IR to the ER formulation upon patient preference, six reported improved convenience of use (75%).

In contrast to the rapid peak-to-trough fluctuations observed with the IR formulation, the ER formulation demonstrates a more linear pharmacokinetic profile. The attenuation of fluctuations may help reduce systemic toxicity and decrease the likelihood of concentration-related adverse effects.^11^ In the present study, although two of the four patients who were initiated directly on LEV ER discontinued treatment due to adverse effects, it remains unclear whether these outcomes were directly attributable to the pharmacological formulation. Notably, all patients who were switched from LEV IR to LEV ER due to sleep disturbances reported symptomatic improvement. Nevertheless, further clinical investigations with larger sample sizes are needed to validate these observations.

Psychiatric disorders and depression represent a significant challenge for both physicians and patients during the treatment of epilepsy patients treated with epileptic drugs. In a large cohort of epilepsy patients, Duman and colleagues identified psychiatric disorders in 26% of 1,329 patients (n=338) and emphasized that both depression and psychotic disorders were prominent negative prognostic factors.^12^ The psychiatric effects of antiepileptic drugs cover a wide spectrum: while some agents may increase the risk of behavioral disturbances, depression, anxiety, attention deficits, and suicidality, others may exert mood-stabilizing or anxiolytic effects. Levetiracetam has been shown in studies to cause psychiatric and cognitive adverse effects, including mood disorders, anxiety, and attention deficits, in both pediatric and adult populations.^13^,^14^ A systematic review assessing real-world data on the frequency of behavioral adverse events associated with brivaracetam, levetiracetam, perampanel, and topiramate in patients with epilepsy underscored that a history of psychiatric disorders in adult epilepsy patients represents a strong predictor for the development of psychiatric and behavioral adverse events during ASM treatment, emphasizing the necessity of incorporating psychiatric comorbidities into therapeutic decision-making.^15^

Moreover, data regarding the side effects of anti-seizure medications across various studies have exhibited considerable inconsistencies. While some studies have demonstrated that LEV has a similar or superior profile to other antiseizure medications with regard to these outcomes, other studies have indicated an increased prevalence of psychiatric and behavioral adverse effects in patients treated with LEV IR.^16^-^19^ This may be attributed to the possibility that patients are at an elevated risk of developing psychiatric side effects associated with levetiracetam, potentially linked to a genetic predisposition and a history of psychiatric disorders.^20^,^21^

Despite these findings, data comparing the psychiatric safety of LEV ER and LEV IR formulations remain limited. A meta-analysis conducted by Richy et al. revealed that patients treated with LEV ER (1000 mg once daily) exhibited a lower incidence of psychiatric disorders compared to those treated with LEV IR (500 mg twice daily).^5^ The authors attributed this difference to the more stable plasma concentration profile associated with LEV ER, which may reduce the risk of central nervous system side effects related to peak concentrations.^22^ In this study, among the four patients initiated on LEV ER based on dosing preference, one had a pre-existing diagnosis of bipolar disorder; notably, no psychiatric side effects were observed in this individual during follow-up. Of the 36 patients who were switched from levitiracetam IR to LEV ER, 26 were switched due to psychiatric side effects, two were discontinued with the recommendation of a psychiatrist on the basis of lack of benefit, and one was continued. Of the 26 patients who underwent a medication change due to mood symptoms, 24 patients (88.4%) reported a regression of their symptoms during psychiatric follow-up. These findings indicate that the use of LEV ER in our study population was associated with a lower incidence of psychiatric side effects than LEV IR. Furthermore, the implementation of drug changes due to adverse effects was effective in reducing the prevalence of reported complaints. Additionally, 12 of the 36 patients who transitioned to LEV ER had a history of psychiatric medication use. Among these, 10 individuals (83.3%) reported improvements in psychiatric symptoms following the formulation change, while two patients experienced no benefit, leading to discontinuation in one case. These outcomes reinforce the importance of considering psychiatric comorbidities when selecting ASMs and suggest that switching to the ER formulation may help alleviate psychiatric symptoms in some patients.

These findings should be interpreted with caution given the study’s limitations. The retrospective design carries an inherent risk of recall and selection bias. Furthermore, patients were not randomized or blinded to their treatment, and no validated questionnaires or structured interviews were used to assess psychiatric or behavioral side effects. Consequently, the data were derived entirely from patient-reported outcomes, which introduces a risk of information bias.

Despite these limitations, the observed reduction in seizure frequency and psychiatric side effects after transitioning from LEV IR to LEV ER is consistent with previously published findings. The pharmacokinetic stability of the ER formulation may therefore offer a meaningful clinical advantage, especially in patients vulnerable to mood disturbances.

## CONCLUSION

This study demonstrated that extended-release levetiracetam significantly reduced seizure frequency and improved treatment compliance in patients with epilepsy. Switching from immediate-release to extended-release formulations was particularly beneficial for those with psychiatric side effects or sleep disturbances. The ER formulation was associated with fewer psychiatric adverse effects, consistent with prior evidence. Although limited by its retrospective design and small sample size, our findings suggest that LEV ER may represent a safer and more convenient therapeutic option in clinical practice.

### Author’s Contributions:

**ZO, FE, GG:** Conceptualization. **ZO, FAC, FE, GG:** Data curation.

**FE, GG:** Formal analysis, Validation. **ZO, FAC:** Investigation. **FE, GG:** Methodology.

**GG:** Project administration, Supervision. **FAC:** Software. **ZO:** Visualization.

**ZO, FE, GG:** Writing - Original draft, review & editing.

All authors contributed to the study conception, design, data collection, analysis, and manuscript preparation.

**ZO** is responsible for the integrity of the work as a whole, including the accuracy of the data and analyses.

All authors have read and approved the final manuscript and agree to be accountable for all aspects of the work.
